# Heterologous expression of an α-amylase inhibitor from common bean (*Phaseolus vulgaris*) in *Kluyveromyces lactis* and *Saccharomyces cerevisiae*

**DOI:** 10.1186/s12934-017-0719-4

**Published:** 2017-06-15

**Authors:** Stephanie Brain-Isasi, Alejandro Álvarez-Lueje, Thomas Joseph V. Higgins

**Affiliations:** 1 Drug Analysis Laboratory, Facultad de Ciencias Químicas y Farmacéuticas, Universidad de Chile, Santiago, Chile; 2 CSIRO Agriculture and Food, GPO Box 1700, Canberra, ACT 2601 Australia

**Keywords:** Phaseolamin, α-Amylase inhibitor, αAI, Common bean, *Phaseolus vulgaris*, Yeast heterologous expression, *Kluyveromyces lactis*, *Saccharomyces cerevisiae*

## Abstract

**Background:**

Phaseolamin or α-amylase inhibitor 1 (αAI) is a glycoprotein from common beans (*Phaseolus vulgaris* L.) that inhibits some insect and mammalian α-amylases. Several clinical studies support the beneficial use of bean αAI for control of diabetes and obesity. Commercial extracts of *P. vulgaris* are available but their efficacy is still under question, mainly because some of these extracts contain antinutritional impurities naturally present in bean seeds and also exhibit a lower specific activity αAI. The production of recombinant αAI allows to overcome these disadvantages and provides a platform for the large-scale production of pure and functional αAI protein for biotechnological and pharmaceutical applications.

**Results:**

A synthetic gene encoding αAI from the common bean (*Phaseolus vulgaris* cv. Pinto) was codon-optimised for expression in yeasts (*αAI*-*OPT*) and cloned into the protein expression vectors pKLAC2 and pYES2. The yeasts *Kluyveromyces lactis* GG799 (and protease deficient derivatives such as YCT390) and *Saccharomyces cerevisiae* YPH499 were transformed with the optimised genes and transformants were screened for expression by antibody dot blot. Recombinant colonies of *K. lactis* YCT390 that expressed and secreted functional αAI into the culture supernatants were selected for further analyses. Recombinant αAI from *K. lactis* YCT390 was purified using anion-exchange and affinity resins leading to the recovery of a functional inhibitor. The identity of the purified αAI was confirmed by mass spectrometry. Recombinant clones of *S. cerevisiae* YPH499 expressed functional αAI intracellularly, but did not secrete the protein.

**Conclusions:**

This is the first report describing the heterologous expression of the α-amylase inhibitor 1 (αAI) from *P. vulgaris* in yeasts. We demonstrated that recombinant strains of *K. lactis* and *S. cerevisiae* expressed and processed the αAI precursor into mature and active protein and also showed that *K. lactis* secretes functional αAI.

**Electronic supplementary material:**

The online version of this article (doi:10.1186/s12934-017-0719-4) contains supplementary material, which is available to authorized users.

## Background

Phaseolamin or α-amylase inhibitor (αAI) is a seed storage glycoprotein present in most wild and cultivated common beans (*Phaseolus vulgaris* L) [1]. It inhibits some mammalian and insect α-amylases, but not the endogenous plant enzyme [2–5]. Bean αAI is synthesised as a preproprotein that undergoes glycosylation at Asn residues and proteolytic processing to become the active form, comprising α- and β- subunits [4, 6–9]. Different cultivars of *P. vulgaris* contain slightly different forms of αAI exhibiting variation in their primary structure, their N-glycans as well as some heterogeneity in the processing of the C-terminal region of the β- subunit. They all retain their inhibitory activity of α-amylases [9].

The functional protein αAI is composed of non-covalently linked α- and β- subunits [10]. In *P. vulgaris*, the αAI precursor undergoes post-translational modifications while transiting the secretory pathway. In addition to N-linked glycosylations, three proteolytic cleavages occur during the maturation of αAI. Removal of the signal peptide occurs in the endoplasmic reticulum (ER). Two carboxy-terminal cleavages occur in the vacuole: the first cleavage at Asn^239^ removes a short carboxy terminal peptide (propeptide), and the second cleavage is catalysed by a bean vacuolar processing enzyme from the family of legumains (VPE) at Asn^77^, resulting in the formation of α- and β- subunits [4, 6–9]. After the proteolytic cleavage at Asn^77^, the asparagine residue is removed from the C-terminus of the α-subunit by a presumed carboxypeptidase in the bean [9].

The inhibition of mammalian α-amylases by αAI reduces starch metabolism and the production of simple sugars, thus its use may be beneficial in helping control diabetes mellitus and obesity in humans [11–13]. The clinical use of bean αAI has been discredited because of the ineffective commercial products released in the early 1980s promoted as starch-blockers [12, 14, 15]. Most of these dietary supplements contained protein concentrates of bean seeds with a low concentration of αAI but with significant levels of antinutritional and potentially toxic compounds, such as phytohemagglutinin (PHA) and trypsin inhibitors, in addition to an active endogenous α-amylase [13].

In contrast to the impure and inefficient commercial formulations, partially purified αAI from beans with increased specific activity reduces starch digestion in human duodenum and remains stable in gastrointestinal secretions [16]. Further studies reported that purified αAI also delayed the digestion and absorption of intestinal glucose, reduced postprandial hyperglycaemia and insulin levels in diabetic and non-diabetic subjects, supporting the notion of the beneficial effect of αAI for the control of diabetes [17–20]. More recent studies using commercial extracts of *P. vulgaris* standardising the content of α-amylase inhibitor determined that many still contain antinutritional impurities, and those with a reduced level of lectins and trypsin inhibitors also exhibit a lower specific activity αAI [21–25]. Proteins naturally present in bean seeds such as PHA-L and PHA-E and the lectin-like proteins called arcelins [4, 9, 26] are difficult to remove during purification of αAI from bean seeds [9, 27]. In an attempt to decrease the amount of lectins in bean seeds, a null PHA bean line obtained by backcrossing has been used as raw material for commercial αAI formulations, but seeds from this line still contain trypsin inhibitors [24, 26]. Given that ingestion of trypsin inhibitors increases the secretion of trypsin and α-amylase by the pancreas [28, 29], their presence in commercial bean extracts are likely to affect the activity and efficacy of αAI.

To date there is no efficient method to recover highly pure and fully functional αAI from bean seeds nor an appropriate expression system for the production of purified recombinant αAI. Here we report the heterologous expression of αAI in yeast, using *Kluyveromyces lactis* and *Saccharomyces cerevisiae* as hosts.

## Methods

### Strains and culture media

*Escherichia coli* DH5-α was used as host for plasmid propagation and was grown in LB medium. Yeast strains used in this work were *K. lactis* wild type GG799 (*MAT*α) and protease deficient strains YCT389 (*MAT*α Δ*yps1p*), YCT390 (*MAT*α Δ*yps7p*), YCT569 (*MAT*α Δ*pfam00026*), and YCT598 (*MAT*α Δ*Bar1p*) (purchased from NEB, Ipswich, MA, USA) [30] and *S. cerevisiae* YPH499 (*MAT*a *ura3*-*52 lys2*-*801_amber ade2*-*101_ochre trp1*-Δ*63 his3*-Δ*200 leu2*-Δ*1*) [31]. Yeast strains were routinely grown in standard YPD medium. Cultures were induced using YPCGal medium (1% yeast extract, 2% peptone, 2% casaminoacids, 2% galactose and 2 mM ammonium sulphate, supplemented with B complex vitamins [32]) at 28 °C for 72–96 h at 250 rpm. Selection of recombinant strains of *K. lactis* was based on growth on YCB agar plates containing 5 mM acetamide [33]. *S. cerevisiae* transformants were selected by their growth in yeast synthetic drop-out medium without uracil agar (SC-ØU).

### Construction of expression vectors and yeast transformation

**Fig. 1:**
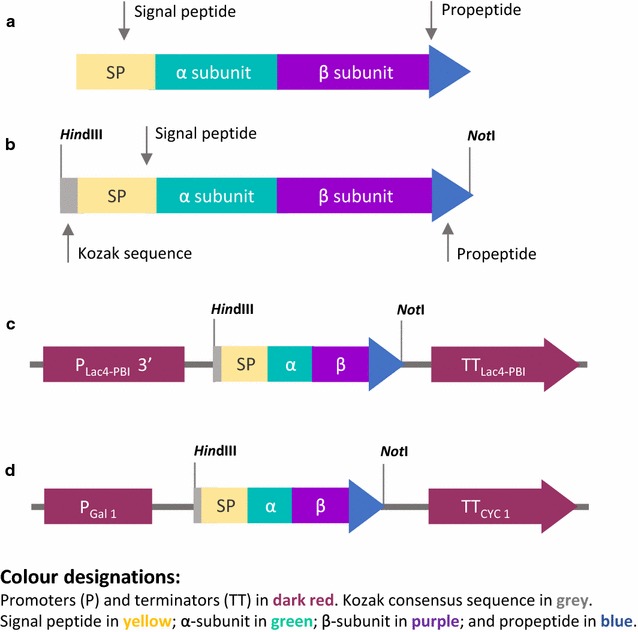
Schematic representation of the gene constructs used for αAI production. Diagrams of **a** the native *αAI* gene from cv. Pinto comprising signal peptide (SP), α- and β- subunits and the propeptide sequences; **b** the synthetic *αAI*-*OPT* gene, optimised for expression in two yeasts, including a Kozak consensus sequence and flanked by *Hind*III and *Not*I restriction sites. Diagrams of constructs **c** pKLAC2-αAI-OPT and **d** pYES2-αAI-OPT. P_Lac4-PBI_ and P_Gal1_ correspond to promoters Lac4-PBI and Gal1 in pKLAC2 and pYES2, respectively. TT_Lac4-PBI_ and TT_CYC_ correspond to 3′ terminators Lac4-PBI and CYC in pKLAC2 and pYES2, respectively

The synthetic gene *αAI*-*OPT* was synthesised by GenScript (Piscataway, NJ, USA) using the coding sequence (CDS) of αAI from *P. vulgaris* cv. Pinto (Fig. 1a, as Accession AY603476) and optimised for yeast codon usage bias, including a Kozak consensus sequence AAAAAG upstream of the start codon and flanking restriction sites *Hind*III and *Not*I (Fig. 1b). *αAI*-*OPT* gene was cloned into plasmids pKLAC2 (NEB) and pYES2 (Invitrogen, Carlsbad, CA, USA) at restriction sites *Hind*III and *Not*I, and the resulting vectors were verified by sequence analysis. In construct pKLAC2-αAI-OPT, the *αAI*-*OPT* CDS replaced the αMF secretion domain from vector pKLAC2. Restriction endonucleases and T4 DNA ligase were purchased from NEB and standard DNA techniques were performed as described by Sambrook [34]. Chemically competent yeast cells were prepared using the LiAc/SS carrier DNA/PEG method [35]. *K. lactis* wild type and protease deficient strains were transformed with 1 µg of the integrative cassette from vector pKLAC2-αAI-OPT (Fig. 1c) previously linearised with *Sac*II [33]; and *S. cerevisiae* YPH499 was transformed with plasmid pYES2-αAI-OPT (Fig. 1d).

### Isolation and manipulation of nucleic acids

Plasmid DNA was extracted from *E. coli* and yeasts using QIAPrep^®^ Spin Miniprep Kit (Qiagen, Hilden, Germany) and genomic DNA from yeasts was isolated using DNeasy^®^ Blood and Tissue Kit (Qiagen). Spheroplasts were obtained by incubating yeast cells with 1 U of lyticase (Sigma-Aldrich, St. Louis, MO, USA) for 1 h at 37 °C. *αAI*-*OPT* gene was detected by PCR amplification of a 160 bp amplicon using primers *OPT Fwd* (TATGACGGTCAAAACGCTGA) and *OPT Rev* (GCGGAGAAACCAACTCTGAC). Multicopy integration of cassettes was determined by PCR using primers 2, 3 and 5 [33]. Oligonucleotides were purchased from Sigma-Aldrich (Castle-Hill, NSW, Australia). Samples for nucleotide sequencing were purified using QIAquick^®^ PCR Purification Kit (Qiagen). DNA sequencing reactions were amplified using primers *KLSQ Fwd* (GCGGATAACAAGCTCAAC) and *KLSQ Rev* (TTATCGCACAAGACAATC) and BigDye Terminator v3.1 Cycle Sequencing Kit (Applied BioSystems, Foster City, CA, USA), and further analysed in an automatic DNA sequencer ABI 3130xl Genetic Analyser (Applied BioSystems) at the John Curtin Medical Research School, Australian National University (Canberra, ACT, Australia).

### Cassette copy-number analysis by q-PCR

Q-PCR analysis was performed using FastStart SYBR Green Master without Rox (Roche Life Science, Rotkreuz, Switzerland) in the CFX96™ real-time PCR detection system (BioRad). The *αAI*-*OPT* gene was detected using primers *OPT Fwd*/*OPT Rev*, and the single-copy gene, *KlACT1,* was amplified using primers *KlACT1 Fwd*/*KlACT1 Rev* [36]. The conversion of mass concentration of the vector to copy concentration was as described by Whelan et al. [37]. Experiments were performed in triplicate using DNA from three independent biological samples.

### Screening of recombinant αAI

Transformed colonies were transferred onto a replica plate and each colony was grown in 600 µL of YPCGal media in a 96-well microplate at 28 °C for 72–96 h at 250 rpm. Cleared supernatants (200 µL) were loaded into a dot blot apparatus connected to a vacuum pump. Pure Pinto-αAI [2] in sterile YPCGal (1 ng/µL) was used as positive control. Cleared YPCGal inoculated with untransformed cells (200 µL) and sterile YPCGal (200 µL) were used as negative controls. The nitrocellulose membrane was probed with a rabbit anti-αAI polyclonal antibody (1:10,000 dilution), and then probed with HRP-conjugated anti-rabbit secondary antibody (1:1000 dilution, Promega Corp. Madison, WI, USA). Protein antibody complexes were made visible using Pierce ECL Western Blotting Substrate detection reagent (Thermo-Fisher Scientific, Inc., Waltham, MA, USA) and autoradiography film (Kodak Biomax X-ray film). The relative amount of protein in dots was estimated by densitometry using ImageJ Software [38].

### Protein analysis by SDS PAGE and western blot

Proteins from culture supernatants were precipitated using TCA/acetone and resuspended in ammonium bicarbonate buffer (100 mM ammonium bicarbonate, pH 8.2, 10% DMSO). Proteins from yeast cells were extracted using YeastBuster Protein Extraction Reagent (Novagen, Darmstadt, Germany), 50 μL of 100 × THP, protease inhibitors (2 μM PMSF, 30 μg leupeptin, 5 μg pepstatin A) and 1 U of DNAse. Pure Pinto-αAI was used as positive control for SDS-PAGE (3 μg) and western blots (200 ng plus 3 μg of BSA).

Samples were analysed on NuPAGE^®^ Novex^®^ 10% Bis–Tris Pre-Cast Protein Gels (Invitrogen), loaded in MES Running Buffer and stained with Coomassie G-250. For western blotting, separated proteins were electro-transferred onto a nitrocellulose membrane (0.2 µm Hybond-C, Amersham Biosciences) and probed as described for antibody dot blot. Membranes were stained with Amidoblack prior to probing with the αAI antibody [39]. Protein concentration was determined by Bradford assay [40], using Coomassie Protein Assay (Thermo-Fisher Scientific) and BSA (0–7 µg/µL) as standard.

### α-Amylase activity assay

α-Amylase inhibitory activity was determined using Megazyme Ceralpha reagent as substrate (nonreducing-end blocked *p*-nitrophenyl-α-d-maltoheptaoside, BPNPG7; plus glucoamylase and α-glucosidase; Megazyme International, Ireland) and porcine pancreatic α-amylase (#A6255 Type I-A, Sigma Aldrich), as described by Sarmah et al. [41]. Inhibitory activity was calculated as follows: 1 U of α-amylase activity is the amount of enzyme that generates 1 μmol/min of *p*-nitrophenol under assay conditions; inhibition units or units of α-amylase inhibition (IU) is the amount of inhibitor that reduces the activity of α-amylase by 50% [2]. Experiments were performed in triplicate and results were expressed as the average ± standard deviation. Prior to inhibitor activity assays of culture supernatants, samples were partially purified by DEAE-Sepharose to remove interfering compounds from culture media.

### Statistical analysis

The significance of differences between measurements was analysed using Student’s unpaired *t* test. Values are reported as the average ± standard deviation. Differences in means were considered significant if *P* < 0.05.

### Purification of recombinant αAI

Induced culture supernatants were dialysed against 0.02% sodium azide solution for 24 h at 4 °C and freeze-dried. The lyophilised powder was resuspended in 5 mL of 10 mM citrate/phosphate buffer (10 mM disodium phosphate; 50 mM citric acid; pH 8.0) and incubated with 5 g of DEAE-Sepharose^®^ CL-6B (Pharmacia Biotech, Sweden) for 1 h at 4 °C. Unbound protein was eluted with 10 mL of 10 mM citrate/phosphate buffer. Fractions containing protein bound to the resin were eluted with 5 mL of 250 mM NaCl solution in 10 mM citrate/phosphate buffer, using a glass sintered-funnel (porosity #2) connected to a vacuum pump by a Büchner flask. Samples were dialysed against 0.02% sodium azide solution for 24 h at 4 °C and freeze-dried. Pre-purified samples were incubated with 5 mL of α-amylase-Sepharose in 20 mL of stabilization buffer (15 mM succinic acid, 20 mM CaCl_2_, 0.5 M NaCl, pH 5.6) for at least 2 h at 37 °C. Unbound protein was eluted with two washes of 15 mL of stabilization buffer and protein bound to the resin was eluted with two washes of 15 mL of releasing buffer (0.15 M citric acid, pH 3.2) and pH of samples was immediately increased to 5.6 using 0.1 M NaOH. Fractions were dialysed against 0.02% sodium azide solution for 24 h at 4 °C, freeze-dried and stored at 4 °C for further analyses. All eluted fractions were analysed by Bradford assay and Ceralpha assay. Resin α-amylase-Sepharose was prepared as previously described [4].

### Identification of recombinant αAI by MS

Protein (25 µg) was loaded on SDS-PAGE and bands corresponding to the non-processed (approximately 35 kDa) and processed forms (a combination of α- and β- subunits of αAI, with a molecular mass between 15 and 20 kDa) were excised from the gel. Samples were submitted to Australian Proteome Analysis Facility (Macquarie University, Sydney, NSW, Australia), digested with trypsin and loaded onto a capillary LC system coupled to an MS/MS instrument (1D Nano LC ESI MS/MS) as described by Atack et al. [42]. Tryptic fragments were identified using Mascot Server software (Matrix Science Inc., MA, USA) using the *Phaseolus_vulgaris* database.

## Results

### Construction of yeast expression vectors and screening for secretion of recombinant αAI

**Fig. 2:**
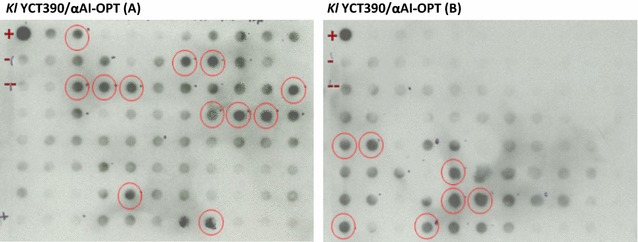
Antibody dot blot screening of culture supernatants of *K. lactis* YCT390 with *αAI*-*OPT*. Cleared supernatants (200 µL) of transformed colonies of *K. lactis* YCT390/αAI-OPT. Pure Pinto-αAI (200 ng) used as positive control (+). As negative controls, cleared culture supernatant (200 µL) of untransformed strain YCT390 (−) and culture media YPCGal (200 µL) without cells (− −). *Circled dots* correspond to *dots* with a signal intensity equivalent to that of positive control

In preliminary experiments we assessed the expression of αAI in *K. lactis* and *S. cerevisiae* using the native gene isolated from beans (shown schematically in Fig. 1a), but no protein expression could be detected (data not shown). We used the amino acid sequence of αAI from cv. Pinto to design and synthesise a gene optimised for yeast codon usage (Fig. 1b). The DNA sequence of the yeast-optimised synthetic gene called *αAI*-*OPT* is shown in Additional file 1: Figure S1. *αAI*-*OPT* was cloned downstream of the galactose-inducible promoters LAC4_PBI_ and Gal1 in plasmids pKLAC2 and pYES2, respectively (Fig. 1c, d). *K. lactis* wild type and protease deficient strains were transformed with the integrative cassettes derived from construct pKLAC2-αAI-OPT and cells were grown in selective YCB agar plates containing acetamide. In addition, *S. cerevisiae* YPH499 was transformed with episomal plasmid pYES2-αAI-OPT and cells were grown in yeast SC-ØU agar plates. Since a variable number of plasmid cassettes can be integrated in the *K. lactis* genome with consequent differences in the *αAI*-*OPT* gene dosage, some variability in the expression of recombinant αAI was to be expected. Therefore, we performed a preliminary screening for the expression of recombinant αAI over a large number of colonies instead of first sorting colonies by genetic analysis. Colonies (186) from each transformed strain of *K. lactis* and *S. cerevisiae* were grown and induced in micro-scale cultures, and the cleared culture supernatants were screened by protein dot blot using an antibody to αAI. In some cultures of *K. lactis* YCT390 (circled dots in Fig. 2), the signal intensity was equivalent to 200 ng of pure Pinto-αAI. Cultures from recombinant *S. cerevisiae* YPH499 and other strains of *K. lactis* (GG799, YCT389, YCT569 and YCT589) showed a signal intensity equivalent to that of the negative controls (see Additional file 2: Figure S2).

### *Saccharomyces cerevisiae* YPH499 pYES2-αAI-OPT clones expressed αAI intracellularly

**Fig. 3:**
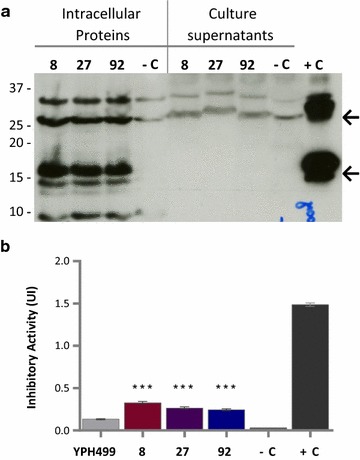
Western blot and α-amylase inhibitory activity of proteins from selected colonies of *S. cerevisiae* YPH499 with *αAI*-*OPT*. **a** Western blot of proteins (25 µg) from culture supernatants and lysed-cells of colonies 8, 27 and 92 transformed with *αAI*-*OPT*; the parent strain YPH499 transformed with empty plasmid pYES2 was used as negative control (−C) and pure Pinto-αAI (200 ng) was used as positive control (+C). Molecular markers are shown in kilodaltons at *left*. **b** Inhibitory activity against porcine pancreatic α-amylase of lysed-cell extracts (100 µg of protein) of *S. cerevisiae* YPH499/pYES2-αAI-OPT colonies 8, 27 and 92. Parent strain YPH499 transformed with empty plasmid pYES2 was used as a negative control (−C) and pure Pinto-αAI (200 ng) was used as a positive control (+C). Results are expressed as inhibition units (IU). Data from triplicate experiments were expressed as the average ± standard error. ***P < 0.05 as compared to *S. cerevisiae* YPH499/pYES2

Although dot blot screening of *S. cerevisiae* YPH499/pYES2-αAI-OPT cultures showed no indications of secreted αAI, plasmid DNA from 10 randomly selected clones was analysed by PCR to screen for the gene. All colonies were positive for the gene (see Additional file 3: Figure S3). To determine whether these clones expressed recombinant αAI, we analysed the proteins in both the culture supernatants and in the lysed cells of three randomly selected clones (8, 27 and 92) by western blot. Immunoreactive polypeptides of the expected size of processed αAI were detected in the intracellular contents of the three recombinant colonies of *S. cerevisiae* YPH499 but were not detected in the culture supernatants (Fig. 3a). Furthermore, no bands corresponding to αAI precursor were detected in either the lysed cells or in the secreted fraction (Fig. 3a).

The intracellular fractions of recombinant clones 8, 27 and 92 of *S. cerevisiae* YPH499 were analysed by Ceralpha assay, using the parent strain with the empty plasmid (YPH499/pYES2) as negative control (Fig. 3b). The inhibition of α-amylase activity by these extracts was very low, but statistically significant (*P* < 0.05) when compared to the negative control.

Although attempts were made to purify the recombinant inhibitor from the lysed cellular contents of *S. cerevisiae* YPH499/pYES2-αAI-OPT, no active inhibitor could be recovered (data not shown).

### αAI was synthesised and secreted by *K. lactis* YCT390/αAI-OPT

**Fig. 4:**
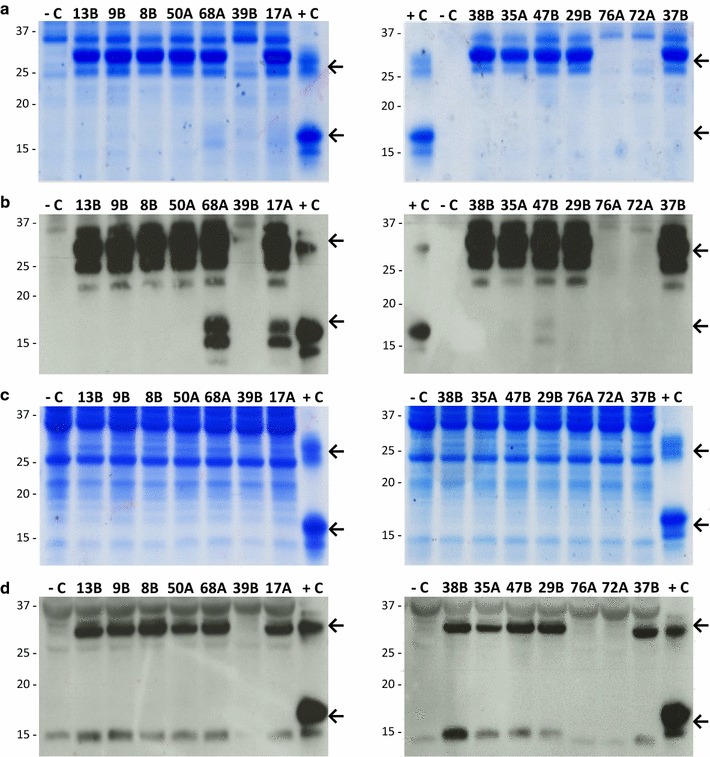
SDS-PAGE and western blots of proteins from selected lines of *K. lactis* YCT390 with *αAI*-*OPT*. **a** SDS-PAGE and **b** Western blot of culture supernatants (TCA-precipitated proteins from 5 mL) of strains 13B, 9B, 8B, 50A, 68A, 39B, 17A, 38B, 35A, 47B, 29B, 76A, 72A and 37B. **c** SDS-PAGE and **d** western blot of lysed-cell contents (25 µg of protein) of strains 13B, 9B, 8B, 50A, 68A, 39B, 17A, 38B, 35A, 47B, 29B, 76A, 72A and 37B. Untransformed strain YCT390 was used as a negative control (−C) and pure Pinto-αAI (200 ng) was used as a positive control (+C). Molecular markers are shown in kilodaltons at *left*

We analysed the proteins from both the lysed-cells and the culture supernatants of *K. lactis* YCT390 transformants by SDS-PAGE and western blot. We chose 10 colonies from among those showing the highest signal intensities in the antibody dot blot as well as four colonies that showed intensities similar to those of negative controls. Eleven of the 14 colonies secreted polypeptides between 15 and 37 kDa (Fig. 4a, b). The top arrows on the right represent the expected molecular mass of αAI precursor; and bottom arrows on the right represent the expected sizes of processed αAI. Three of the 11 colonies (68A, 17A and 47B) secreted two immune-reactive polypeptides within the range of 15 and 20 kDa (Fig. 4b, bottom arrows on the right represent the expected sizes of processed αAI) which exhibited an electrophoretic mobility similar to the Pinto-αAI α- and β- subunits.

The intracellular extracts contained a polypeptide of approximately 32 kDa which co-migrated with the bean αAI precursor but did not contain detectable processed αAI (Fig. 4c, d, top and bottom arrows, respectively).

The genetic analysis of the 14 colonies of *K. lactis* YCT390/αAI-OPT confirmed that the 11 colonies expressing the polypeptides of interest carried the *αAI*-*OPT* gene and also harboured more than one copy of the cassette inserted into the chromosome, whereas non-expressing colonies showed no amplification of the gene (see Additional file 4: Figure S4).

Precursor or processed αAI were not detected in cell lysates and culture supernatants of colonies from the other transformed strains of *K. lactis* (GG799, YCT389, YCT569 and YCT589), although they were positive for *αAI*-*OPT* gene (data not shown).

We measured copy number of *αAI*-*OPT* gene by q-PCR to determine the number of cassettes inserted into the *K. lactis* chromosome. All strains harboured 5–6 copies of *αAI*-*OPT* gene (Table 1).

**Table 1:** Gene copy number and relative processing of recombinant αAI among strains of *K. lactis* YCT390/αAI-OPT

*K. lactis* strain/colony	Gene copy number		Relative processed αAI	Inhibitory activity (%)
	*αAI*-*OPT*	*KlACT1*		
Untransformed YCT390 (negative control)	0	1	–	10.3
YCT390/αAI-OPT 68A	6	1	++++	89.8
YCT390/αAI-OPT 17A	5	1	+++	84.6
YCT390/αAI-OPT 47B	6	1	++	59.2
YCT390/αAI-OPT 29B	6	1	+	49.5
YCT390/αAI-OPT 13B	6	1	–	16.6

Estimated copy number of genes *αAI*-*OPT* and *KlACT1* (control) in genomic DNA of selected strains of *K. lactis* YCT390/αAI-OPT measured by q-PCR. Relative amount of processed recombinant αAI and percentage of inhibitory activity against porcine pancreatic α-amylase among pre-purified extracts of *K. lactis* YCT390/αAI-OPT strains. Untransformed strain YCT390 was used as negative control

### Culture supernatants of *K. lactis* inhibited α-amylase

**Fig. 5:**
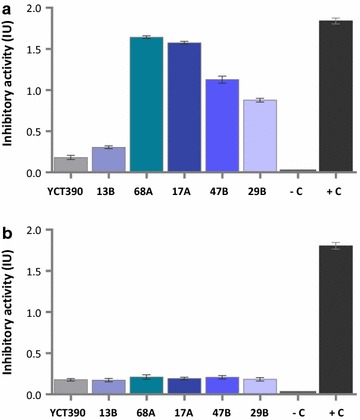
Inhibition of α-amylase by extracts from recombinant strains of *K. lactis*. Inhibition of porcine pancreatic α-amylase by **a** partially purified culture supernatants (300 µg of protein) and **b** lysed-cell extracts (500 µg of protein) from five colonies of *K. lactis* YCT390/αAI-OPT strains 13B, 68A, 17A, 47B and 29B. Untransformed strain YCT390 was used as negative control (−C) and pure Pinto-αAI (200 ng) was used as positive control (+C). Culture supernatants were partially purified with DEAE-Sepharose prior to inhibitor activity assays. Results are expressed as inhibition units (IU). Data from triplicate experiments were expressed as the average ± standard error

To determine whether the polypeptides secreted by selected recombinant strains of *K. lactis* YCT390 were active as α-amylase inhibitors, we assayed partially purified culture supernatants from five strains for activity by Ceralpha assay (Fig. 5a). The culture supernatants were partially purified with DEAE-Sepharose in order to remove compounds present in the culture media interfering with the activity assay. Strains 17A and 68A strongly inhibited α-amylase activity while extracts from 47B and 29B showed an intermediate level of inhibition of the enzyme, namely 57 and 44%, respectively. Strain 13B culture supernatant was much less active, i.e., only slightly more active than the negative control (untransformed YCT390) (Fig. 5a). Activities from lysed-cell extracts of *K. lactis* were not significantly different (*P* < 0.05) to the negative control (Fig. 5b).

### Recombinant αAI was purified from culture supernatants of *K. lactis*

**Fig. 6:**
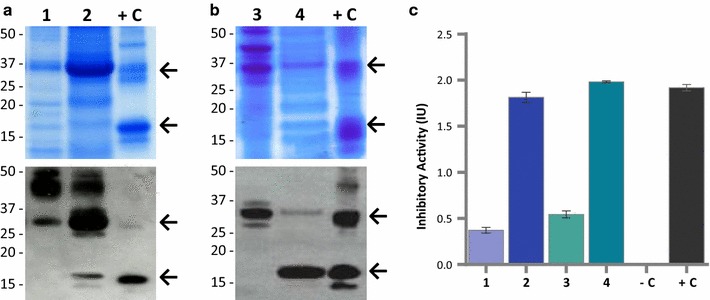
Purification of recombinant αAI from culture supernatants of *K. lactis* YCT390/αAI-OPT. SDS-PAGE and western blot of** a** fractions purified by anion exchange chromatography from *K. lactis* YCT390/αAI-OPT strain 68A: protein unbound to DEAE-Sepharose (*lane 1*), protein bound to DEAE-Sepharose (*lane 2*); and **b** fractions purified by affinity chromatography: unbound proteins to α-amylase-Sepharose (*lane 3*), and proteins bound to α-amylase-Sepharose (*lane 4*). Pure Pinto-αAI (200 ng) was used as a positive control (+C). Molecular markers are shown in kilodaltons at *left*. **c** Inhibition of porcine pancreatic α-amylase by purified fractions containing unbound proteins to DEAE-Sepharose (*lane 1*), bound proteins to DEAE-Sepharose (*lane 2*), unbound proteins to α-amylase-Sepharose (*lane 3*), and bound proteins to α-amylase-Sepharose (*lane 4*). Untransformed strain YCT390 was used as a negative control (−C) and pure Pinto-αAI (200 ng) was used as a positive control (+C). Data from triplicate experiments were expressed as the average ± standard error

The methods to purify αAI from seeds [2–4, 10, 16] were not suitable for the recovery of αAI from culture supernatants possibly because of the composition of YPCGal medium (which has a mixture of hydrolysed peptides, galactose and vitamin B complex). Soluble and functional αAI was recovered from *K. lactis* culture supernatants using a combination of ion exchange and affinity chromatography. The fractions were analysed by SDS-PAGE, western blot and Ceralpha assay (Fig. 6). Electrophoretic analysis of the purified fractions showed that anionic exchange and affinity purification with α-amylase-Sepharose yielded polypeptides with a size between 15 and 20 kDa (Fig. 6a, lane 2, and Fig. 6b, lane 4, bottom arrow points to α- and β- subunits). Activity assay of the fractions obtained by anion-exchange and α-amylase-Sepharose showed that specific inhibitory activity increased following purification (Fig. 6c).

Prior to purification, 300 mL of culture supernatant from *K. lactis* YCT390/αAI-OPT 68A yielded around 0.9 mg protein. After anion-exchange, we recovered about 25% of the protein containing partially purified inhibitor; and after incubation with α-amylase-Sepharose, we recovered approximately 1% of the protein in the culture supernatant as functional αAI (Table 2).

**Table 2:** Purification of recombinant αAI from culture supernatants of *K. lactis* YCT390/αAI-OPT strain 68A

Purification step	Total protein (μg)	Specific inhibitory activity (IU/mg)
Dialysed and freeze-dried culture supernatant	899	ND
Anion-exchange batch-adsorption (DEAE-Sepharose CL-6B)	227	8
Affinity batch-adsorption (α-amylase-Sepharose)	8.45	234

**Fig. 7:**
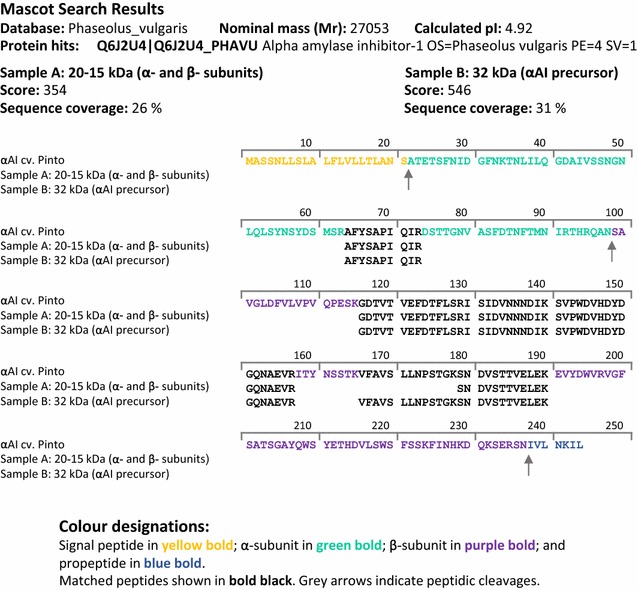
Tryptic peptide matches with Pinto-αAI amino acid sequence (Q6J2U4_PHAVU). Mascot search results and sequence similarity of tryptic fragments obtained from recombinant αAI from purified supernatants of *K. lactis* YCT390/αAI-OPT 68A, compared to amino acid sequence of α- and β- subunits from Pinto-αAI (Q6J2U4_PHAVU). Expected peptide cleavage sites are denoted by *arrows*. Identity threshold score for *P* < 0.05: 13

The identity of the peptides from *K. lactis* YCT390/αAI-OPT 68A was confirmed by 1D Nano LC ESI MS/MS. After electrophoresis, the major bands between 15 and 20 kDa (expected size of α- and β- subunits) were excised from gel together in a single sample (Sample A), and the band of approximately 32 kDa (expected size of αAI precursor) was isolated separately (Sample B). α- and β- subunits of αAI were detected in both of these samples. Processed αAI and the precursor were identified by 5 and 6 distinct tryptic fragments, representing 26 and 31% of coverage, respectively (Fig. 7). No other protein was detected in these major bands.

## Discussion

The α-amylase inhibitor αAI from *P. vulgaris* has been successfully expressed in different plants resulting in functional inhibitor [8, 9]. Nonetheless, the heterologous expression of αAI in a non-plant system has not been reported to date and was not expected to be straight-forward due to the post-translational processing that this protein undergoes during maturation. Nevertheless, using yeasts to express αAI was attractive because of their ability to perform post-translational modifications, as well as their ability to secrete proteins into the culture media, which could facilitate purification.

In preliminary experiments using the plant form of the *αAI* gene to transform strains of *K. lactis* GG799 derivatives and *S. cerevisiae* YPH499, the yeasts expressed the gene transcript but did not express the protein (Brain-Isasi and Higgins, unpublished data). Because recombinant strains of *K. lactis* YCT390 and *S. cerevisiae* YPH499 harbouring the reconstructed gene *αAI*-*OPT* were able to synthesise functional αAI, we conclude that codon usage bias is a limiting step in the heterologous expression of αAI in yeasts.

Here we demonstrated that the native signal peptide of αAI encoded in the synthetic gene *αAI*-*OPT* was recognised by *K. lactis* YCT390 to target the nascent peptide into the secretory pathway. However, we found an unexpected differential trafficking of αAI between recombinant *K. lactis* YCT390 and *S. cerevisiae* YPH499, since recombinant colonies of *K. lactis* YCT390 secreted αAI (precursor and processed polypeptides) into the culture medium and recombinant *S. cerevisiae* YPH499 expressed functional αAI intracellularly that was not secreted. This difference in αAI trafficking between two yeast species resembles the results obtained when PHA-L is expressed in *Pichia pastoris* and *S. cerevisiae* [43, 44]. PHA-L is also a vacuolar glycoprotein from *P. vulgaris* and shows considerable amino acid similarity to αAI, but PHA-L is devoid of the proteolytic processing site at Asn^77^ required for the maturation of αAI [8]. When PHA-L is expressed in *P. pastoris* using its native signal peptide, the signal peptide is proteolytically cleaved and the protein is secreted into the culture medium [44]. In contrast, when PHA-L is expressed in *S. cerevisiae* using its native signal peptide, only about 1% is secreted and most of the protein accumulates in the vacuole because of a vacuolar targeting-determinant in the PHA-L sequence which is recognized by this yeast [43, 45]. Therefore, it is possible that αAI shows a similar behaviour to PHA-L when expressed in *S. cerevisiae.* Like αAI, several other seed proteins require proteolytic cleavage on the carboxyl side of Asn by specific VPEs to make their respective mature forms [46, 47]. To date, no specific Asn-proteases similar to VPEs are known in yeasts. Although yeast Gpi8p shows significant structural similarity to plant VPEs, it is part of the GPI-anchor complex and acts as a transamidase [48].

In other work on yeast overexpression of plant proteins requiring a proteolytic cleavage at an Asn residue, it has been shown that recombinant precursors are not proteolytically processed at the Asn site and if cleaved, this occurs at a different amino acid [49]. For example, when the 2S albumins AL1 and AL3 from soybean (*Glycine max*) were expressed in *P. pastoris*, the subunits were post-translationally processed within the same loop region as in the plant but with some differences in the precise sites [50]. Since recombinant αAI expressed in *S. cerevisiae* YPH499 and *K. lactis* YCT390 was proteolytically processed into α- and β-like subunits, it appears likely that the proteolytic cleavage was not performed at the Asn^77^ residue but this did not affect the inhibitory activity of the protein. Aberrant processing of αAI was observed in transgenic tobacco harbouring a mutation at Asn^77^–Asp^77^, but the specific activity of the processed protein appears to be similar to that of correctly processed αAI [8]. Also, transgenic chickpea-αAI has a subset of molecules in which the α-subunit devoid of Asn^77^ and Ala^76^ retains its inhibitory activity [9].

The integration of cassettes into the chromosome of *K. lactis* regularly produces strains having multiple copies of the vector [33], and it has been reported that gene dosage can affect the yield of some heterologous proteins expressed in yeasts [33, 51, 52]. Here q-PCR data showed that all the *K. lactis* YCT390/αAI-OPT colonies contained an average of six copies of the gene, indicating that there was no correlation between the gene dosage and the differences in processing of αAI. None of the recombinant colonies of *K. lactis* YCT390 contained mutations in the *αAI*-*OPT* gene sequence, and there were no differences in cell growth among the recombinant *K. lactis* YCT390/αAI-OPT colonies when compared to the control strain (data not shown). As previously described for the expression of recombinant phytase in *P. pastoris* [52], it seems that the level of processed αAI produced by *K. lactis* YCT390 was not related to gene dosage, mutations or toxic effects on the host.

When we characterized the heterologous expression of recombinant αAI in *K. lactis* GG799 and in four protease deletion mutants created in GG799 background [30], we found that only recombinant colonies of *K. lactis* YCT390 secreted αAI into the culture media, while in the other strains of *K. lactis* the protein was not detected. The only difference among the *K. lactis* strains assessed in this work lies in the deletion of a single protease from the secretory pathway, since *K. lactis* YCT390 is a mutant deficient in KlYps7p, an aspartyl proteinase localised in the plasma membrane and in the yeast cell-wall [30]. Our results are consistent with the notion that in many cases heterologous proteins are degraded in *K. lactis* due to aspartyl protease, and successful expression is achieved only after using a protease deficient strain [30, 53].

The screening of several hundred transformants is an important bottleneck in yeast expression systems. In our case, the selection of colonies by means of activity or immunodetection was a more efficient method of finding clones that produced a higher level of functional protein than doing an initial genetic screen. Given that the expression profile of αAI was improved in a single *K. lactis* strain (YCT390), and the other *K. lactis* strains that did not express the protein were positive for *αAI*-*OPT* gene, the selection of colonies by antibody dot blot allowed us to reduce the analysis from several hundreds to <14 colonies per strain.

Here we successfully expressed functional αAI in yeasts. However, it has to be noted that while we optimised the codon usage of native bean αAI for yeast expression we did not further modify the coding sequence nor the cultivation conditions. Therefore, it is possible that the amounts of processed protein may be increased further by modifying the cell culturing and induction conditions, using different strategies to enhance proteolytic cleavage, as well as other bioengineering approaches that have been demonstrated to enhance the levels of other heterologous proteins produced by yeasts [54].

## Conclusion

We demonstrated that αAI from *P. vulgaris* can be expressed in two different yeast heterologous systems. We showed that functional αAI can be secreted and purified from culture supernatants by using a two-step chromatographic method. Therefore, our work allows for the production of recombinant αAI and provides a platform for the large-scale production of pure and functional αAI protein for biotechnological and pharmaceutical applications.

## Additional files

**Additional file 1: Figure S1.** Schematic representations of Pinto-αAI gene, synthetic gene and its nucleotide sequence. (**A**) Native *αAI* gene from cv. Pinto comprising signal peptide (SP), α- and β- subunits and the propeptide sequences; (**B**) the synthetic *αAI-OPT* gene, optimised for expression in yeast, including a Kozak consensus sequence and flanked by *Hin*dIII and *Not* Irestriction sites. (**C**) Nucleotide sequence of *αAI-OPT* gene.

**Additional file 2: Figure S2.** Antibody dot blot screening of culture supernatants of *K. lactis* and *S. cerevisiae* with αAI-OPT. Cleared supernatants of transformed colonies of *K. lactis* strains YCT389, YCT390, YCT569, YCT589 and GG799, and *S. cerevisiae* YPH499 transformed with *αAI-OPT* gene. (+) PurePinto-αAI (200ng) used as positive control (+). As negative controls, cleared culture supernatant (200μL) of untransformed strain YCT390 (-) and culture media YPCGal (200μL) without cells (--). Circled dots correspond to dots with a signal intensity equivalent to that of positive control.

**Additional file 3: Figure S3.** Genetic analysis of *S. cerevisiae* YPH499 clones with αAI-OPT. (**A**) Colony PCR of random colonies of *S.cerevisiae* YPH499 transformed with αAI-OPT (primers *OPT Fwd/OPT Rev*). Purified water used as PCR negative control (-C) and plasmid DNA from pYES2-αAI-OPT used as positive control (+C). DNA Ladder 100bp NEB®(M). (**B**)PCR determination of *αAI-OPT* gene on plasmid DNA isolated from transformants of *S.cerevisiae* YPH499 with αAI-OPT (primers *OPT Fwd/OPT Rev*). (**C**) Schematic representation of amplification product with primers *OPT Fwd/OPT Rev.* Plasmid DNA from parent strain transformed with empty plasmid (pYES2) used as negative control. DNA Ladder 1Kb Promega™ (M’).

**Additional file 4: Figure S4.** Genetic analysis of *K. lactis* YCT390 transformants with αAI-OPT. (**A**) Indirect determination by PCR of *αAI-OPT* gene inserted into pKLAC2 using sequencing primers *KLSQ Fwd/KLSQ Rev* in transformants of *K. lactis* YCT3908B, 9B, 13B, 29B, 37B, 38B, 39B, 42B, 47B, 9A, 12A, 17A, 35A, 50A, 68A, 72A and 76A. (**B**) Schematic representation of amplification product (805bp) with primers *KLSQ Fwd/KLSQ Rev*. PCR determination of (**C**) *αAI-OPT* gene fragment with primers *OPT Fwd/OPT Rev*, and (**D**) *KlACT1* gene fragment (single copy gene in *K.lactis* genome) with primers *KLACT1 Fwd/KLACT1 Rev,* in transformants of *K. lactis* YCT39013B, 29B, 37B, 47B, 17A, 35A and 68A.Genomic DNA from untransformed strain YCT390 used as negative control. Purified water used as PCR negative control (-C) and plasmid DNA from pKLAC2-αAI-OPT used as PCR positive control (+C). DNA Ladder 100bp NEB®(M).

## Abbreviations

αAI: α-amylase inhibitor 1
Pinto-αAI: αAI from bean cv. Pinto
PHA-L: phytohemagglutinin-L
αMF: α mating factor
CDS: coding DNA sequence
YPCGal: yeast extract-peptone-casaminoacids-galactose medium
SC-ØU: synthetic drop-out medium without uracil
THP: tris(hydroxypropyl)phosphine
DEAE: diethylaminoethyl/diethylaminoethanol
kDa: kilodalton
M_r_: relative molecular mass
VPE: vacuolar plant enzyme
GPI: glycosylphosphatidylinositol
