# Supplementary material for: Heterologous expression of an α-amylase inhibitor from common bean (Phaseolus vulgaris) in Kluyveromyces lactis and Saccharomyces cerevisiae
Source: Microb Cell Fact. 2017 Jun 15;16:110. doi: 10.1186/s12934-017-0719-4 (PMC5472880; doi:10.1186/s12934-017-0719-4)
Supplement: Supplementary file 1 — Additional file 1: Figure S1. Schematic representations of Pinto-αAI gene, synthetic gene and its nucleotide sequence. (A) Native αAI gene from cv. Pinto comprising signal peptide (SP), α- and β- subunits and the propeptide sequences; (B) the synthetic αAI-OPT gene, optimised for expression in yeast, including a Kozak consensus sequence and flanked by HindIII and Not Irestriction sites. (C) Nucleotide sequence of αAI-OPT gene. [file 12934_2017_719_MOESM1_ESM.pdf]

Figure S1

A

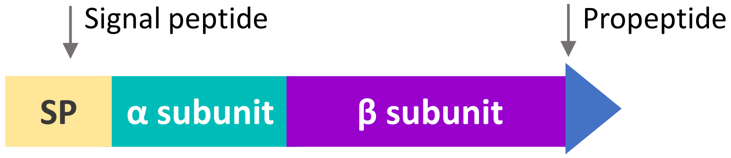

B

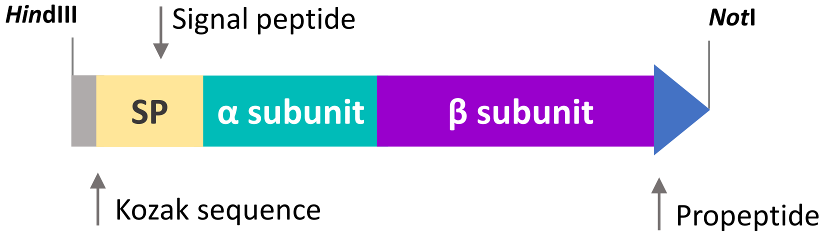

C

aagcttgaaaaa**atggcatcctcaaacttgttgtccttggctttattcttggtcttatta**
accttggcaaactccgctaccgaaacttcctttaacatcgatggttttaacaagaccaat
ttgatcttacaaggtgacgctatcgtttcttcaaacggaaatttgcagttgtcctacaac
agttacgattccatgagtagagccttctattcagctccaattcaaatcagagatagtact
actggtaacgtcgcttcttttgacactaacttcacaatgaacatcagaactcatagacaa
gctaattcagcagttggtttggattttgttttagtcccagttcagcctgagtccaaagga
gatacagtcaccgttgaatttgacactttcttgtcaagaatttctatcgatgttaacaac
aacgacattaagtctgttccttgggacgttcacgattatgacggtcaaaacgctgaggtt
agaatcacctacaattccagtactaaggtcttcgcagtttctttgttgaacccttctaca
ggaaaatcaaatgatgtctccaccactgttgaattggagaaggaagtttacgactgggtc
agagttggtttctccgcaacaagtggagcctatcagtggtcttacgaaacccatgatgtt
ttatcttggtcattttcttcaaaattcattaaccataaggaccagaagtcagagagaagt
aacatcgttttgaataagattttgtaagcggccgc

Restriction sites *Hind*III (5’) and *Not*I (3’) in black bold;
Kozak sequence underlined;
Signal peptide in **yellow bold**; α-subunit in **green bold**; β-subunit in **purple bold**;
and propeptide in **blue bold**.

Figure S1: Schematic representations of Pinto-αAI gene, synthetic gene and its nucleotide sequence. (**A**) Native *αAI* gene from cv. Pinto comprising signal peptide (SP), α- and β- subunits and the propeptide sequences; (**B**) the synthetic *αAI-OPT* gene, optimised for expression in yeast, including a Kozak consensus sequence and flanked by *Hind*III and *Not*I restriction sites. (**C**) Nucleotide sequence of *αAI-OPT* gene.
