# Supplementary material for: Heterologous expression of an α-amylase inhibitor from common bean (Phaseolus vulgaris) in Kluyveromyces lactis and Saccharomyces cerevisiae
Source: Microb Cell Fact. 2017 Jun 15;16:110. doi: 10.1186/s12934-017-0719-4 (PMC5472880; doi:10.1186/s12934-017-0719-4)
Supplement: Supplementary file 2 — Additional file 2: Figure S2. Antibody dot blot screening of culture supernatants of K. lactis and S. cerevisiae with αAI-OPT. Cleared supernatants of transformed colonies of K. lactis strains YCT389, YCT390, YCT569, YCT589 and GG799, and S. cerevisiae YPH499 transformed with αAI-OPT gene. (+) PurePinto-αAI (200ng) used as positive control (+). As negative controls, cleared culture supernatant (200μL) of untransformed strain YCT390 (-) and culture media YPCGal (200μL) without cells (--). Circled dots correspond to dots with a signal intensity equivalent to that of positive control. [file 12934_2017_719_MOESM2_ESM.pdf]

Figure S2

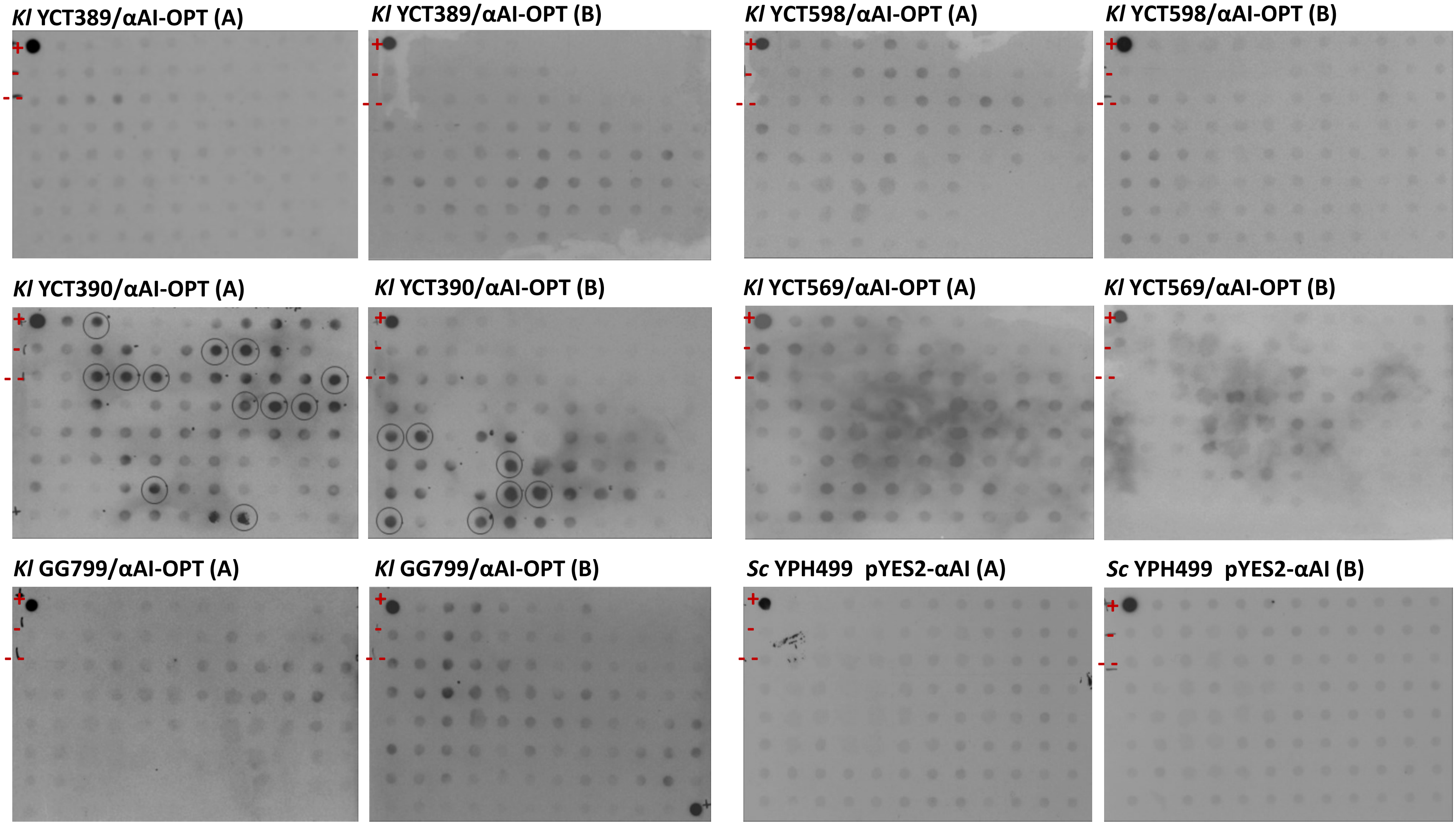

Figure S2: Antibody dot blot screening of culture supernatants of *K. lactis* and *S. cerevisiae* with αAI-OPT. Cleared supernatants of transformed colonies of *K. lactis* strains YCT389, YCT390, YCT569, YCT589 and GG799, and *S. cerevisiae* YPH499 transformed with *αAI-OPT* gene. (+) Pure Pinto-αAI (200 ng) used as positive control (+). As negative controls, cleared culture supernatant (200 µL) of untransformed strain YCT390 (-) and culture media YPCGal (200 µL) without cells (- -). Circled dots correspond to dots with a signal intensity equivalent to that of positive control.
