# Supplementary material for: Heterologous expression of an α-amylase inhibitor from common bean (Phaseolus vulgaris) in Kluyveromyces lactis and Saccharomyces cerevisiae
Source: Microb Cell Fact. 2017 Jun 15;16:110. doi: 10.1186/s12934-017-0719-4 (PMC5472880; doi:10.1186/s12934-017-0719-4)
Supplement: Supplementary file 3 — Additional file 3: Figure S3. Genetic analysis of S. cerevisiae YPH499 clones with αAI-OPT. (A) Colony PCR of random colonies of S.cerevisiae YPH499 transformed with αAI-OPT (primers OPT Fwd/OPT Rev). Purified water used as PCR negative control (-C) and plasmid DNA from pYES2-αAI-OPT used as positive control (+C). DNA Ladder 100bp NEB®(M). (B)PCR determination of αAI-OPT gene on plasmid DNA isolated from transformants of S.cerevisiae YPH499 with αAI-OPT (primers OPT Fwd/OPT Rev). (C) Schematic representation of amplification product with primers OPT Fwd/OPT Rev. Plasmid DNA from parent strain transformed with empty plasmid (pYES2) used as negative control. DNA Ladder 1Kb Promega™ (M’). [file 12934_2017_719_MOESM3_ESM.pdf]

Figure S3

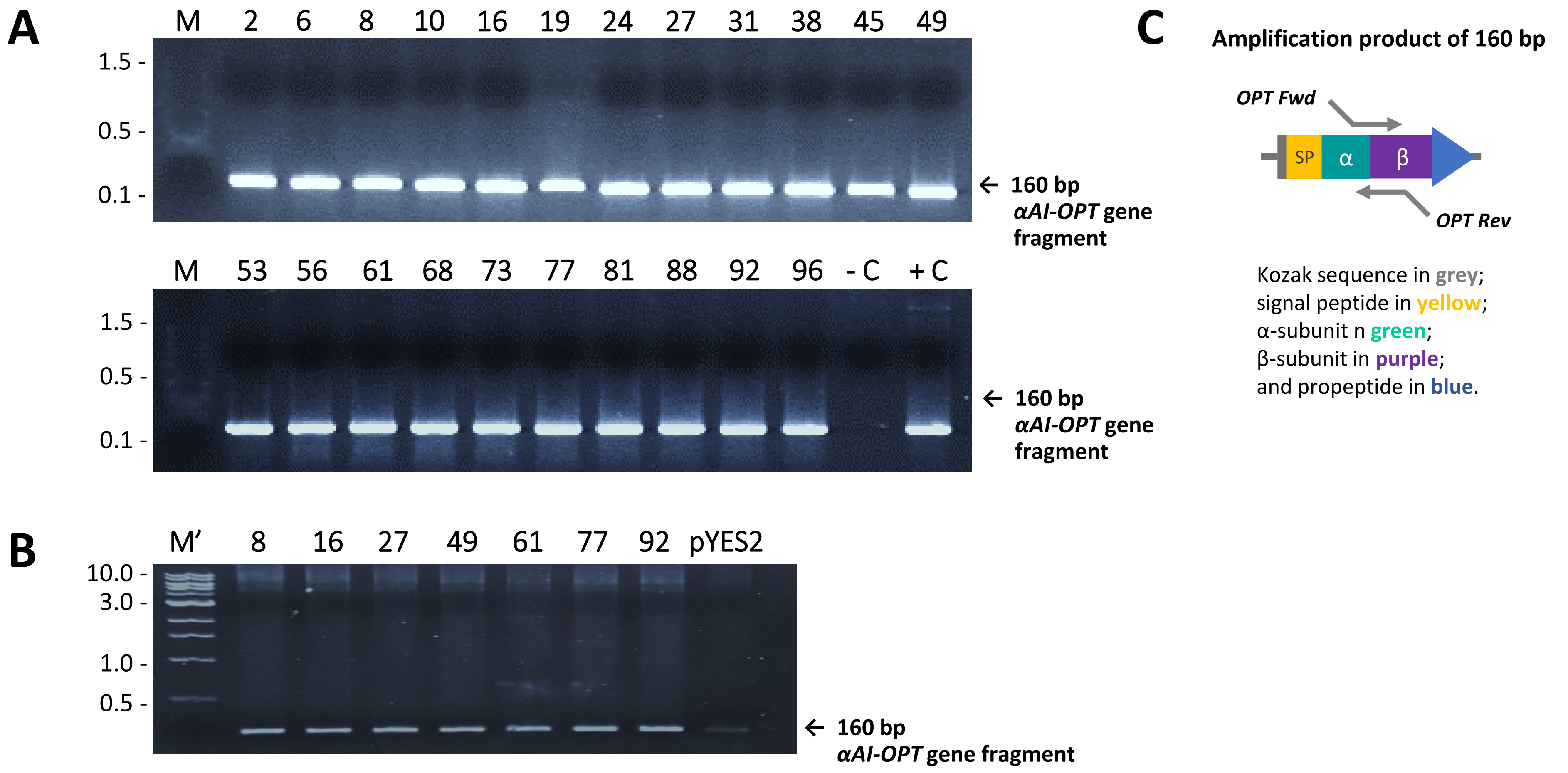

Figure S3: Genetic analysis of *S. cerevisiae* YPH499 clones with αAI-OPT. (**A**) Colony PCR of random colonies of *S. cerevisiae* YPH499 transformed with αAI-OPT (primers *OPT Fwd/OPT Rev*). Purified water used as PCR negative control (-C) and plasmid DNA from pYES2-αAI-OPT used as positive control (+ C). DNA Ladder 100 bp NEB® (M). (**B**) PCR determination of *αAI-OPT* gene on plasmid DNA isolated from transformants of *S. cerevisiae* YPH499 with αAI-OPT (primers *OPT Fwd/OPT Rev*). (**C**) Schematic representation of amplification product with primers *OPT Fwd/OPT Rev.* Plasmid DNA from parent strain transformed with empty plasmid (pYES2) used as negative control. DNA Ladder 1 Kb Promega™ (M').
