# Supplementary material for: Heterologous expression of an α-amylase inhibitor from common bean (Phaseolus vulgaris) in Kluyveromyces lactis and Saccharomyces cerevisiae
Source: Microb Cell Fact. 2017 Jun 15;16:110. doi: 10.1186/s12934-017-0719-4 (PMC5472880; doi:10.1186/s12934-017-0719-4)
Supplement: Supplementary file 4 — Additional file 4: Figure S4. Genetic analysis of K. lactis YCT390 transformants with αAI-OPT. (A) Indirect determination by PCR of αAI-OPT gene inserted into pKLAC2 using sequencing primers KLSQ Fwd/KLSQ Rev in transformants of K. lactis YCT3908B, 9B, 13B, 29B, 37B, 38B, 39B, 42B, 47B, 9A, 12A, 17A, 35A, 50A, 68A, 72A and 76A. (B) Schematic representation of amplification product (805bp) with primers KLSQ Fwd/KLSQ Rev. PCR determination of (C) αAI-OPT gene fragment with primers OPT Fwd/OPT Rev, and (D) KlACT1 gene fragment (single copy gene in K.lactis genome) with primers KLACT1 Fwd/KLACT1 Rev, in transformants of K. lactis YCT39013B, 29B, 37B, 47B, 17A, 35A and 68A.Genomic DNA from untransformed strain YCT390 used as negative control. Purified water used as PCR negative control (-C) and plasmid DNA from pKLAC2-αAI-OPT used as PCR positive control (+C). DNA Ladder 100bp NEB®(M). [file 12934_2017_719_MOESM4_ESM.pdf]

Figure S4

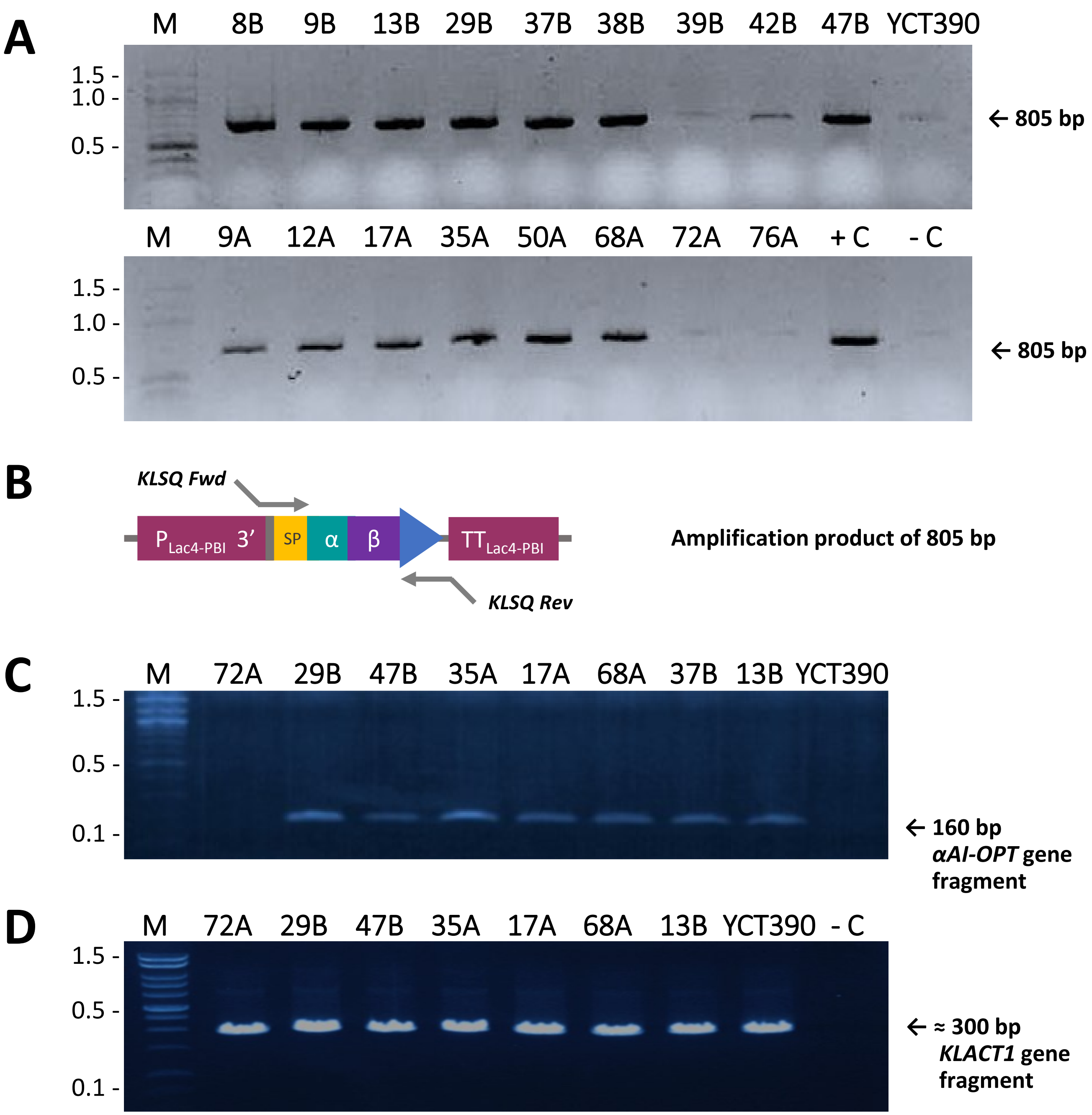

Figure S4: Genetic analysis of *K. lactis* YCT390 transformants with αAI-OPT. (**A**) Indirect determination by PCR of *αAI-OPT* gene inserted into pKLAC2 using sequencing primers *KLSQ Fwd/KLSQ Rev* in transformants of *K. lactis* YCT390 8B, 9B, 13B, 29B, 37B, 38B, 39B, 42B, 47B, 9A, 12A, 17A, 35A, 50A, 68A, 72A and 76A. (**B**) Schematic representation of amplification product (805 bp) with primers *KLSQ Fwd/KLSQ Rev*. PCR determination of (**C**) *αAI-OPT* gene fragment with primers *OPT Fwd/OPT Rev*, and (**D**) *KlACT1* gene fragment (single copy gene in *K. lactis* genome) with primers *KLACT1 Fwd/KLACT1 Rev,* in transformants of *K. lactis* YCT390 13B, 29B, 37B, 47B, 17A, 35A and 68A. Genomic DNA from untransformed strain YCT390 used as negative control. Purified water used as PCR negative control (- C) and plasmid DNA from pKLAC2-αAI-OPT used as PCR positive control (+ C). DNA Ladder 100 bp NEB® (M).
